# Increasing dietary roughage equivalent from 7 to 12% during the final 28 days on feed does not impact growth performance but may improve ruminal health in feedlot steers

**DOI:** 10.1093/tas/txag068

**Published:** 2026-05-20

**Authors:** Forest L Francis, Federico Podversich, Warren C Rusche, Zachary K Smith

**Affiliations:** Department of Animal Science, South Dakota State University, Brookings, SD 57007, United States; Department of Animal Science, South Dakota State University, Brookings, SD 57007, United States; Department of Animal Science, South Dakota State University, Brookings, SD 57007, United States; Department of Animal Science, South Dakota State University, Brookings, SD 57007, United States

**Keywords:** carcass, efficiency, finishing, roughage level, ruminal health

## Abstract

The objective was to determine if increasing roughage equivalent (RE) from 7% to 12% (17.5% to 20.5% dietary neutral detergent fiber on a DM basis) in the last 28 days of fattening could improve rumen health parameters and decrease the frequency of liver abscesses without compromising productive efficiency or carcass characteristics. Charolais × Angus crossbred steers [*n* = 64; *n* = 8 pens; initial shrunk body weight (SBW) = 633 kg ± 35.1 kg] were allocated to dietary treatment in a completely randomized design. All steers were fed a common 7% [dry matter (DM) basis] roughage equivalent diet (17.5% neutral detergent fiber [NDF] DM basis) where the roughage sources were based upon corn silage and hay for the 58 d prior to study initiation. The treatments involved increasing the dietary forage concentration from 7% to 12% for the final 28 days on feed, as follows: (1) No diet change (7R) and (2) 5% (DM basis) roughage equivalent addition to 7R via grass hay during the final 28 DOF to increase the dietary NDF to 20.5% DM basis (12R). Steers were finished on treatment diets for 28 d prior to harvest. No differences (*P* ≥ 0.19) were observed for any growth performance, efficiency, or carcass trait measures except for marbling scores. Marbling score differed (*P* = 0.04) between treatments with 12R steers exhibiting 5% greater marbling than 7R steers. A treatment × DOF interaction (*P* < 0.01) was observed for minutes ruminating per day. Daily minutes ruminating was increased (*P* ≤ 0.05) for 12R on d 1, 2, and 20 and tended to be increased (*P* ≤ 0.10) for 12R on d 10, 13, 24, and 25. Rumenitis score nor was liver abscess prevalence was influenced by dietary treatment. Inclusion of an additional 5% RE to cattle diets during the final 28 DOF can be a management strategy to maintain ruminal health without sacrificing growth performance or carcass quality, but does not appear to reduce incidence of liver abscessation.

## Introduction

Liver abscesses continue to be a problem at the feedyard and beef processor because of decreased live performance and carcass characteristics with any severity of abscessed liver. Liver abscesses have been associated with acidosis induced rumenitis from feeding diets high in readily fermentable starch (ie high-moisture, steam-flaked, or dry-rolled grain) and low in roughage (Bryant and Jennings 2022). Increased roughage levels in the diet can reduce the prevalence of liver abscesses because of starch dilution in the diet, increased rumination time, and increased ruminal scratch factor, which can all improve rumen health and mitigate the risk of acidosis induced ruminitis (Amachawadi and Nagaraja 2022). Previous literature indicates that minor severity liver abscesses can heal and will leave lesions or scars on the liver (Jensen et al. 1947; Garcia et al. 1974; Itabisashi et al. 1987). Jensen et al. (1947) and Itabisashi et al. (1987) reported that liver abscesses may heal between 40 d to 70 d following initial detection, however, because of number of days between detection periods in these studies, abscess healing may be accomplished more rapidly.

The current dogma of feeding and finishing cattle is to grow cattle on higher roughage, lower energy diet and eventually transition the cattle to a higher starch, high energy diet to fatten cattle until harvest. Late feeding period roughage inclusion for mitigation of liver abscesses and decreased risk of acidosis has been only finitely researched because of high price of roughage sources and anticipated loss of cattle performance by decreasing starch content of diets. Bartle and Preston (1991) and Loerch and Fluharty (1998) both reported that feeding a high-concentrate diet early in the finishing phase and adding roughage back into the diet in the late finishing period caused no adverse effects on performance and cut liver condemnation rates were cut in half. These studies evaluated adding roughage back into the diet for longer periods of time (100 + d); if similar results could be obtained by adding roughage to finishing diets for shorter periods of time, then cattle feeders might be more accepting of changing diets late in the feeding period and not incurring dramatic increases in ration costs or having to substantially alter feed storage and milling capabilities. Thus, the objective was to determine if increasing roughage equivalent (RE) from 7% to 12% (17.5% to 20.5% dietary neutral detergent fiber on a DM basis) in the last 28 days of fattening could improve rumen health parameters and decrease the frequency of liver abscesses without compromising productive efficiency or carcass characteristics. It was our hypothesis that by including additional roughage in the diet, ruminal health would improve, and minor severity liver abscesses would have time to resolve resulting in a decrease in total liver abscesses, all without compromising growth efficiency or character traits.

## Materials and methods

All procedures involving the use of animals in these experiments were approved by the South Dakota State University Institutional Animal Care and Use Committee (Approval #2207-037E).

### Dietary treatments

This study used 4 replicate pens per treatment with each pen containing 8 steers (*n* = 32 steers/treatment). Each pen was assigned to one of two dietary treatments in a completely randomized design. All steers were fed a common 7% [dry matter (DM) basis] roughage equivalent diet (17.5% neutral detergent fiber [NDF] DM basis) where the roughage sources were based upon corn silage and hay for the 58 d prior to study initiation. The treatments involved increasing the dietary forage concentration from 7% to 12% for the final 28 days on feed, as follows:

1. No diet change. Steers remained on 7% roughage equivalent (DM basis) that contained 17.5% dietary NDF on a DM basis (7R)
2. 5% roughage equivalent (DM basis) addition to diet via grass hay during the feeding of ractopamine hydrochloride to provide 20.5% dietary NDF on a DM basis (12R).

All diets were fortified with vitamins and minerals to exceed nutrient requirements for confinement fed growing steers (NASEM 2016). No tylosin phosphate was fed during the experiment and monensin sodium (Rumensin-90; Elanco Animal Health, Greenfield, IN) was fed at 32 mg/kg of complete diet [dry matter (DM) basis]. Ractopamine hydrochloride (RH) was fed for the 28 d study period at 300 mg/steer-d^−1^ and was provided in a ground corn carrier that contained 2 g/kg RH.

### Cattle feeding and management

Sixty-four steers [initial shrunk body weight (BW) = 633 kg ± 35.1 kg] were sourced from an unrelated study conducted at the South Dakota State University Ruminant Nutrition Center for use in the current study. Prior to study initiation, all steers consumed a common 93% concentrate diet (7R; Table 1) that contained 7% roughage equivalent from corn silage and grass hay. The corn silage was harvested on 18 August 2021; (estimated yield [as-is] = 40.4 t/ha; actual DM = 38.3%; actual crude protein [CP], NDF, and starch = 7.7, 40.3, and 34.0%, respectively [DM basis]). A self-propelled forage harvester (John Deere 9800i; Deere and Company, Moline, IL) was used for harvesting and kernel processing. Kernel processing was achieved by narrowing the reverse sawtooth processing rollers (John Deere XSTREAM KP; Deere and Company) to 2 mm. Corn silage processing scores (starch proportion of native sample retained below a 0.45 mm sieve) was 71.0%. Corn silage was stored in a concrete bunker silo (bunker face 3.6 m × 3.0 m) that was covered with an oxygen exclusion barrier. Hay was processed using a tub grinder equipped with a 101.6 mm screen (Haybuster, DuraTech Industries International Inc., Jamestown ND), the hay was assessed to using the Penn State Particle Separator and material retained was 42.0%, 22.2%, 14.8%, and 21.0% on the 19-mm, 8-mm, 4-mm sieves, and pan, respectively. All steers in the current study remained in their home pens from the previous study to not disturb pen hierarchy. On 30 June 2022 steers were processed where an individual BW was recorded. Following processing event, pens were randomized to treatments and received treatment diets (Table 1). Steers remained in home pens until d 28 of study when they were processed again and individual BW was recorded to obtain a final weight for study resolution.

**Table 1:** Ingredients and analyzed chemical composition of dietary treatments fed to finishing beef steers based on 7% or 12% roughage equivalent during the final 28 d before harvest.

Ingredients^1^	Dietary treatments	
	7R	12R
**Dry rolled corn, %**	68.10	63.09
**Liquid supplement^2^, %**	4.94	4.94
**DDGS, %**	14.92	14.92
**Corn silage^3^, %**	10.03	10.03
**Grass hay, %**	2.01	7.02
**Dry matter, %**	73.55	73.63
**Crude protein, %**	13.38	13.43
**Neutral detergent fiber, %**	17.53	20.57
**Acid detergent fiber, %**	8.47	10.33
**Ether extract, %**	3.87	3.79
**Ash, %**	5.35	5.74
**Net energy for maintenance, Mcal/kg**	2.07	2.02
**Net energy for gain, Mcal/kg**	1.42	1.37

1 All ingredients on dry matter basis except dry matter.

2 Liquid supplement contained (dry matter basis): 41.86% crude protein, 38.38% non-protein nitrogen, 0.95 Mcal/kg net energy for maintenance, 0.66 Mcal/kg net energy for gain, 0.91% ether extract, 10.89% Ca, 0.32% P, 7.00% K, 0.22% Mg, 6.03% NaCl, 3.07% Na, 0.33% S, 4.23 ppm Co, 199.88 ppm Cu, 11.99 ppm I, 15.08 ppm EDDI, 83.16 ppm Fe, 304.81 ppm Mn, 2.90 ppm Se, 664.59 ppm Zn, 44065.04 IU/kg Vitamin A, 440.66 IU/kg Vitamin E, and 638.75 mg/kg monensin sodium.

3 The corn silage was harvested on 18 August 2021; (estimated yield [as-is] = 40.4 t/ha; actual DM = 38.3%; actual crude protein [CP], NDF, and starch = 7.7, 40.3, and 34.0%, respectively [DM basis]). A self-propelled forage harvester (John Deere 9800i; Deere and Company, Moline, IL) was used for harvesting and kernel processing. Kernel processing was achieved by narrowing the reverse sawtooth processing rollers (John Deere XSTREAM KP; Deere and Company) to 2 mm. Corn silage processing scores (starch proportion of native sample retained below a 0.45 mm sieve) was 71.0%. Corn silage was stored in a concrete bunker silo (bunker face 3.6 m × 3.0 m) that was covered with an oxygen exclusion barrier.

Steers were fed in 58 m^2^ concrete pens with 7.6 m of linear bunk space and equipped with continuous flow concrete water troughs that were shared between pens within block. Individual ingredient samples were collected weekly, and DM was calculated following drying in a 60°C forced air oven until no weight change to calculate dry matter intake (DMI). Proximate analysis of ingredients was conducted weekly; DM [method no. 935.29 (AOAC 2012)], N [method no. 968.06 (AOAC 2016); Rapid Max N Exceed, Elementar Americas, Inc., Ronkonkoma, NY], and ash [method no. 942.05 (AOAC 2012)]. Ether extract (EE) content analysis was conducted for MDGS using an Ankom Fat Extractor (XT10; Ankom Technology, Macedon, NY); tabular EE values were used for the remainder of the ingredients (NASEM 2016). Acid detergent fiber (ADF) and neutral detergent fiber (NDF) percentages were estimated to be 3% and 9%, respectively, for DRC; fiber content analysis for all other ingredients was conducted as described by (Goering and Van Soest, 1970). Actual diet formulation based upon weekly DM determination and feed batching record with tabular energy values according to Preston (2016).

Steers were fed twice daily at 0800 h and 1500 h and intake was managed according to a slick bunk management system allowing ad libitum access to feed, with minimal day to day variation in feed deliveries. Feed was manufactured in a commercial stationary mixer wagon (volume 2.26 m^3^; Roto-Mix 84-8, Roto-Mix LLC, Dodge City, KS) with a scale resolution of 0.45 kg. Weighed feed was delivered to individual pens via a modified chain driven delivery wagon.

### Rumination and activity tracking

All steers (*n* = 64) were fitted with an Allflex eSense Flex tag (Allflex Livestock Intelligence; Merck & Co., Rahway, NJ) 14 d prior to study initiation to track daily rumination (minutes). Tags were placed in the middle one-third of the steers right ear and remained in place for the entirety of the study. Rumination data was transmitted to a receiver and downloaded into the Allflex Heatime Pro+ (Allflex Livestock Intelligence; Merck & Co.) platform and raw data was downloaded for each steer at study termination. Data used for analysis began on d -7 at 0000 h and ended on d 28 at 2400 h. Day -7 to d -1 values were averaged and used as baseline rumination measures for statistical analysis. Tags were removed during the final processing event prior to shipping.

### Carcass characteristics and rumen health collection

On study d 28 (28 July 2022), steers were weighed for final BW determination; the same afternoon all steers were transported 238 km to a commercial beef processor for harvest the following morning. Steers were comingled at the time of shipping and remained this way until harvest. Trained individuals entered the slaughter facility and individual visual identification tags were recorded. Additionally, all steers had been previously administered a radio frequency identification tag; these tags were recorded at the beef processor via an Allflex RS420NFC Series Stick Reader (Allflex USA; Merck & Co). Livers were visually evaluated to determine health according to the Elanco Liver Check System (Elanco, Greenfield, IN). Rumen health was evaluated via an adapted scoring system adapted from Bigham and McManus (1975) where rumen lesions and papillae clumping were evaluated on a 0-10 scale (0: no rumen lesions or papillae clumping, 10: 100% of the rumen had lesions or papillae clumping). Because of the small sample size in this study rumens were grouped based on rumen health score into the following categories: 0 = Healthy, 1 to 4 = Mild, 5 to 10 = Severe. Rumens were scored following removal of all contents and washing of the rumen wall. Packer identification tags were recorded to ensure individual carcasses could be traced back to live steer identification. Hot carcass weight was obtained via plant printouts. Following chilling, all carcasses were ribbed for USDA-AMS grading; quality and yield grade attributes were obtained with camera grading. Dressing percentage was calculated as: (HCW ÷ final shrunk BW) × 100. Yield grade was calculated according to the USDA regression equation (USDA 2017). Estimated empty body fat (EBF) percentage and final BW at 28% EBF (AFBW) were calculated from observed carcass traits (Guiroy et al. 2002).

### Growth performance calculations

Growth was calculated on a shrunk live basis. All steers were weighed individually at initial processing and on d 28. Steers were weighed in a hydraulic squeeze chute mounted on top of load cells (scale readability ± 0.45 kg). Live growth performance was based upon initial BW shrunk 4% and final BW shrunk 4%. Average daily gain (ADG) was calculated as the difference between initial BW shrunk 4% and final BW shrunk 4% divided by days on feed. Feed conversion efficiency (G : F) was calculated from ADG ÷ DMI.

### Efficiency of dietary NE utilization calculations

Applied energetics measures (observed dietary NE and the ratio of observed-to-expected dietary NE) were assessed for the feeding period based upon live growth performance. Live performance based dietary NE was calculated from daily energy gain (EG; Mcal/d): EG = (ADG)^1.097^ × 0.0557 W^0.75^; where W is the mean equivalent shrunk BW [kg; (NASEM 2016)] from median feeding SBW (MBW) and AFBW calculated as: [MBW × (478 ÷ AFBW), kg; (NASEM 2016)]. Median feeding shrunk BW was calculated as: (Final SBW + Initial SBW) ÷ 2. Adjusted final BW was calculated via the equations described in Guiroy et al. (2002). Maintenance energy (EM) was calculated by the equation: EM = 0.077 × MBW^0.75^ (NASEM 2016). Dry matter intake is related to energy requirements and dietary NE for maintenance (NEm; Mcal/kg) according to the following equation: DMI = EG ÷ (0.877NEm—0.41), and can be resolved for estimation of dietary NEm by means of the quadratic formula $x=\frac{-b\pm \sqrt{b^{2}-4\mathrm{ac}}}{2c}$, where a = 0.41EM, b = −0.877EM + 0.41DMI + EG, and c = −0.877DMI (Zinn et al. 2008). Dietary NE for gain (NEg) was derived from NEm using the following equation: NEg = 0.877NEm—0.41 (Zinn et al. 2008). Observed-to-expected (O : E) NEm and NEg were a ratio of performance-based dietary NE to tabular dietary NE values (Preston 2016). Net energy for gain intake (Mcal/d; RE) was calculated as: Feed available for gain, kg (FFG) × Diet NEg (Mcal/kg), where FFG = (DMI—DMI for maintenance), and DMI for maintenance = ((0.077 × MBW^0.75^) ÷ (Diet NEm, Mcal/kg). Energy density of live weight gain (GED) was calculated as: RE ÷ ADG; units for GED are (Mcal/kg) to compare caloric content of live weight gain (Smith et al. 2018).

### Statistical analysis

Growth performance, efficiency of dietary NE utilization, and continuous carcass data were analyzed as a completely randomized design using the GLIMMIX procedure of SAS 9.4 (SAS Inst. Inc; Cary, NC). Pen was considered the experimental unit and treatment was a fixed effect in model analysis. Least square means were generated with the LSMEANS option of SAS and significance was determined at *P* ≤ 0.05 and tendencies to differ were observed at 0.05 < *P* ≤ 0.10.

Rumination activity data was analyzed using the GLIMMIX procedure of SAS 9.4. Pen was considered the experimental unit. The model included the fixed effects of treatment, week, the treatment by week interaction and baseline minutes ruminating as a covariate. Additionally, block was included as a random effect. For the conformation of the repeated measures split plot structure, a second random was included using with pen within treatment as the subject. The covariance structure utilized was Toeplitz, which was selected based on the smallest AICC in the model. Least square means were generated with the LSMEANS option of SAS, and differences between treatments within week were obtained using the slice by week option. Significance was determined at *P* ≤ 0.05 and tendencies were observed at 0.05 < *P* ≤ 0.10.

Categorical carcass data and rumen health data were analyzed with individual steer as the experimental unit. The fitted model was analyzed with the Fisher’s exact test via the FREQ procedure of SAS 9.4 to determine differences between treatments. Significance was determined at *P* ≤ 0.05 and tendencies were observed at 0.05 < *P* ≤ 0.10.

## Results

The results for live basis growth performance and efficiency of dietary net energy utilization are reported in Table 2. No differences (*P* ≥ 0.19) were observed for final SBW, ADG, DMI, G : F, observed NE_m_, observed NE_g_, observed-to-expected NE_m_, observed-to-expected NE_g_, RE, or GED.

**Table 2:** Live basis growth performance of beef steers fed 7% (7R) or 12% (12R) roughage equivalent from ground hay during the final 28 d prior to harvest.

Item	Dietary Treatments		SEM	*P*-value
	7R	12R		
**Pens, *n***	4	4	–	–
**Steers, *n***	32	32	–	–
**Growth performance^1^**				
**Initial body weight, kg**	632	634	3.0	0.66
**Final body weight, kg**	680	683	6.2	0.80
**Average daily gain, kg**	1.73	1.74	0.131	0.92
**Dry matter intake, kg**	11.46	12.06	0.290	0.19
**G : F**	0.151	0.144	0.0083	0.59
**Dietary NE utilization**				
**Observed NE_m_, Mcal/kg**	2.25	2.16	0.063	0.36
**Observed NE_g_, Mcal/kg**	1.56	1.48	0.055	0.36
**Observed-to-expected NE_m_**	1.09	1.07	0.031	0.69
**Observed-to-expected NE_g_**	1.10	1.08	0.040	0.75
**NE_g_ intake^2^, Mcal/d**	9.39	9.74	0.375	0.53
**Gain energy density^3^, Mcal/kg**	5.47	5.67	0.307	0.65

1 Growth performance was calculated on a 4% shrunk live basis.

2 NE_g_ Intake (Mcal/d) was calculated as: Feed available for gain, kg (FFG) × Diet NEg (Mcal/kg), where FFG = (DMI—DMI for maintenance), and DMI for maintenance = ((0.077 × MBW^0.75^) ÷ (Diet NEm, Mcal/kg).

3 Gain energy density (GED) was calculated as: NE_g_ Intake ÷ ADG; units for GED are (Mcal/kg) to compare caloric content of live weight gain (Smith et al. 2018).

Continuous carcass characteristics are reported in Table 3. No differences (*P* ≥ 0.35) were observed for HCW, dressed yield, ribeye area, 12th rib fat thickness, calculated yield grade, or calculated empty body fat percentage. Marbling score differed (*P* = 0.04) between treatments with 12R steers exhibiting 5% greater marbling in the ribeye.

**Table 3:** Carcass data of steers fed 7% (7R) or 12% (12R) roughage equivalent during the final 28 d prior to harvest.

Item	Dietary Treatments		SEM	*P*-value
	7R	12R		
**Pens, *n***	4	4	–	–
**Steers, *n***	32	32	–	–
**Carcass characteristics**				
**Hot carcass weight, kg**	439	438	5.6	0.87
**Dressed yield^1^, %**	64.55	64.14	0.295	0.37
**Ribeye area, cm^2^**	97.56	99.40	1.287	0.35
**12^th^ rib fat thickness, cm**	1.72	1.70	0.043	0.73
**Marbling score^2^**	519	544	6.9	0.04
**Calculated yield grade**	3.40	3.27	0.088	0.36
**Calculated empty body fat^3^, %**	32.69	32.44	0.272	0.54

1 Calculated as: (hot carcass weight ÷ final body weight, shrunk 4%) × 100).

2 Marbling scores: 400 = Small00 (Minimum for USDA Choice), 500 = Modest00 (Minimum for USDA Premium Choice).

3 Empty body fat (EBF) = 17.76207 + (4.68142 × FT) + (0.01945 × HCW) + (0.81855 × QG) − (0.06754 × LMA); FT = fat thickness, cm; HCW = hot carcass weight, km; QG = quality grade (Select = 4, Low Choice = 5, Average Choice = 6, High Choice = 7, Low Prime = 8); LMA = longissimus muscle area, sq cm (Guiroy et al. 2002).

Categorical carcass data is reported in Table 4. No difference was observed for USDA Quality grade distribution (*P* = 0.58) or USDA yield grade distribution (*P* = 0.31). Additionally, rumen and liver health data are reported in Table 5. No differences between treatments were observed for rumenitis score (*P* = 0.31) or liver abscess prevalence (*P* = 1.00).

**Table 4:** Categorical carcass data of beef steers fed 7% (7R) or 12% (12R) roughage equivalent from ground hay during the final 28 d prior to harvest.

Item	Dietary Treatments		*P*-value
	7R	12R	
**Steers, *n***	32	32	
**Quality grade**			
**Select, %**	9.38	9.38	0.58
**Low choice, %**	43.75	25.00	
**Average choice, %**	21.88	34.38	
**High choice, %**	21.88	28.13	
**Prime, %**	3.11	3.11	
**Yield grade**			
**1, %**	3.12	0.00	0.31
**2, %**	25.00	25.00	
**3, %**	53.13	68.75	
**4, %**	18.75	6.25	

**Table 5:** Ruminal and liver health of beef steers fed 7% (7R) or 12% (12R) roughage equivalent from ground hay during the final 28 d prior to harvest.

Item	Dietary Treatments^1^		*P*-value
	7R	12R	
**Steers, *n***	32	32	
**Rumenitis score^1^**			
**Healthy, %**	37.93	45.16	0.31
**Mild, %**	41.38	48.39	
**Severe, %**	20.69	6.45	
**Liver abscess**			
**No abscess, %**	90.63	90.63	1.00
**Abscess, %**	9.37	9.37	

1 Rumen health was evaluated via an adapted scoring system adapted from Bigham and McManus (1975) where rumen lesions and papillae clumping was evaluated on a 0–10 scale (0: no rumen lesions or papillae clumping, 10: 100% of the rumen had lesions or papillae clumping). Rumens were grouped based on rumen health score into the following categories: 0 = Healthy, 1 to 4 = Mild, 5 to 10 = Severe.

**Figure 1:**
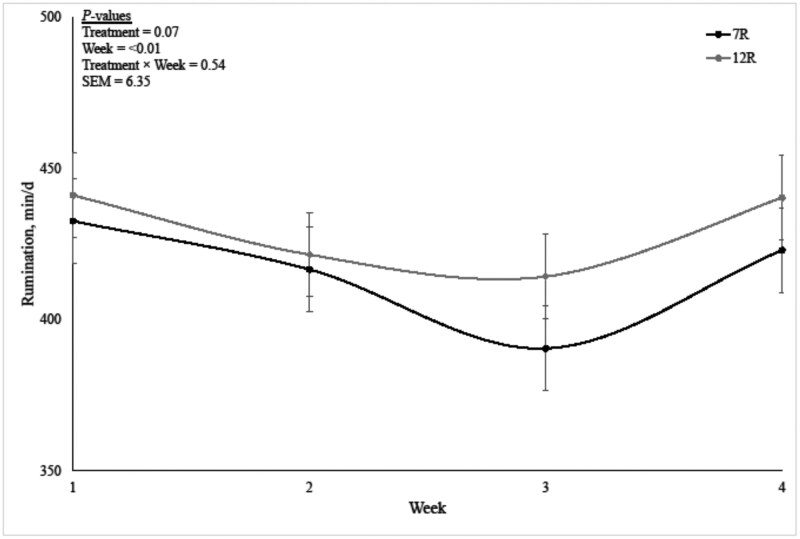
Weekly minutes ruminating for steers fed 7% (7R) or 12% (12R) roughage equivalent diets during the final 28 days on feed.

Daily rumination minutes for the feeding period are reported in Fig. 1, while daily activity (min/d) and rumination expressed as min per kg of DMI or NDFI are presented in Table 6. No interactions were observed between treatment and week for any of the variables evaluated (*P* > 0.51). Daily activity and daily rumination expressed in min per day differ across weeks (*P* < 0.01), while rumination expressed in min per kg of DMI or NDFI did not differ across weeks (*P* > 0.23). Level of roughage fed did not affect daily activity (*P* = 0.86). Rumination expressed in min per day tended to be greater for steers fed the 12R diet than for steers fed the 7R diets by 13.6 min/d (*P* = 0.07; 429.3 vs 415.7 min/d, for 12R vs 7R, respectively). Yet, rumination expressed in min per kg of DMI did not differ between treatments (*P* = 0.57). Inversely, rumination expressed in min per kg of NDF consumed tended to be lower for the steers fed 12R than for steers fed with the 7R diet (*P* = 0.06; 171.4 vs 209.2 min/kg NDFI, for 12R vs 7R, respectively).

**Table 6:** Weekly activity and rumination of beef steers fed 7% (7R) or 12% (12R) roughage equivalent from ground hay during the final 28 d prior to harvest.

	Treatment (Trt)											
	7R				12R							
	Week				Week					*P-*value		
	1	2	3	4	1	2	3	4	SEM	Trt	Week	Trt × Week
**Activity, min/d**	326.9	316.7	330.8	310.9	331.6	319.5	327.5	313.4	5.63	0.86	<0.01	0.66
**Rumination, min/kg dry-matter intake**	36.5	36.9	35.9	37.2	36.7	35.6	34.5	35	1.27	0.57	0.37	0.51
**Rumination, min/kg neutral detergent fiber intake**	208	212.2	203.7	212.8	176.5	173.8	165.5	169.6	6.85	0.06	0.23	0.61

## Discussion

In cattle fed finishing diets, intake is likely controlled by metabolic factors as compared to bulk fill of the gastrointestinal tract (Galyean and Defoor 2003). When roughage levels in finishing diets are increased, it has been reported that cattle will increase intake to maintain energy intake to reach a ‘metabolic fill’ (Galyean and Defoor). By increasing roughage in the diet from 7% to 12%, steers in the current study followed this same trend. Steers on the 12R diet had 5% greater DMI than 7R steers and had similar ADG as steers on the 7R diet. Data from this study would indicate that steers on the 12R diet consumed more feed to maintain energy intake as previously reported in the literature. To validate this result, daily NE_g_ intake and GED were calculated to determine if increased DMI truly resulted in increased energy intake. There was no difference between 7R and 12R for NE_g_ intake or GED, indicating that by increasing roughage 5%, DMI was stimulated to a point where energy intake was not sacrificed, and energy density of live weight gain was maintained. Because DMI was increased with no change in ADG, a 4.5% decrease in G : F was observed for 12R cattle compared to 7R cattle.

Steers on the 12R diet consumed 83% more roughage (DM basis) over the study period than steers on the 7R diet. This increase in roughage consumption was reflected in a 3.3% increase in daily average minutes ruminating for 12R compared to 7R steers. Increasing dietary concentration of dry roughages has been shown to increase chewing time and saliva production, thus increasing total buffering from saliva which can help neutralize and dilute ruminal acids (Owens et al. 1998). With increased rumination noted in the 12R steers, these steers should have increased buffering from saliva compared to 7R steers. Additionally, by increasing the percentage of dietary roughage, starch content of the diet is diluted, thus decreasing the dietary concentration of highly fermentable carbohydrates which should improve ruminal pH levels.

Another factor that is considered important in promoting rumination or rumen papillae development is the physical scratching effect of more lignified fiber sources (Russell 2017). While the experimental and physiological support of this theory is difficult to find, previous research in sheep and feedlot cattle have shown the benefits of physical scratch in the rumen via dietary roughage and dosage with plastic pot scrubbers in the rumen (Ørskov et al. 1979; Loerch 1991). Feedlot steers fed a 100% concentrate diet that were ruminally dosed with either 4 or 8 plastic pot scrubbers had similar growth performance as steers fed an 85% concentrate diet (roughage from corn silage) and had improved growth performance compared to steers on a 100% concentrate diet with no pot scrubbers (Loerch 1991). Additionally, steers fed the 85% concentrate diet and those receiving pot scrubbers had rumen papillae that were dense and uniform in size, whereas steers fed the 100% concentrate diet without pot scrubbers showed evidence of papillary sloughing, impaction of hair and feed, clumping, and irregular size (Loerch 1991). In the present study, by increasing dietary roughage 5%, apparent improvements in rumen health were observed at slaughter. While not statistically significant (*P* = 0.31), 12R had a 19% increase in steers with healthy rumens and a 69% decrease in steers with severe ruminitis or clumping compared to 7R. While improvements in rumen health would seemingly lead to a decrease in liver abscess prevalence, no difference was observed between treatments for occurrences of liver abscesses. It should be noted that the overall prevalence of liver abscesses in this study was low (9%) for finishing cattle in the Midwest United States which have a normal prevalence around 20% to 30% (Grimes 2022). Further research investigating late period dietary roughage inclusion in feedlot cattle should be conducted with cattle that have greater potential for liver abscesses to determine if minor severity abscesses have time to heal as rumen health improves.

With equivalent energy intake and energy density of live weight gain along with improved rumen health, 12R steers exhibiting similar carcass characteristics compared to 7R steers is a logical outcome. A notable difference between the two groups is the slight decrease in dressed yield for 12R steers compared to 7R. Steers on the 12R diet had numerically higher final SBW and minutely lower hot carcass weight compared to 7R steers which led to a numerically decreased dressed yield percentage. Because 12R steers were fed a higher roughage diet and had increased DMI compared to 7R steers it is likely that gut fill was greater for 12R cattle and was reflected in a numerically lower dressing percentage for cattle in this treatment. Marbling score differed (*P* = 0.04) between treatments with 12R steer exhibiting a 5% increase in marbling score compared to 7R steers. While a statistical difference was observed, marbling score treatment averages for both 7R and 12R steers fell within the Average Choice quality grade. The distribution of USDA quality and yield grades did not statistically differ between treatments; however, some notable differences were observed between treatments for these variables. A 40% increase in the number of carcasses grading Average Choice or better was observed for 12R steers compared to 7R steers. Additionally, an 83% decrease in the number of yield grade 4 carcasses was observed for 12R compared to 7R steers. During the 28-d feeding period, NE_g_ intake and GED were similar for both treatments of cattle but numerically increased for 12R. Historical literature is divided on differential utilization of acetate, glucose, and lactate in subcutaneous (SC) and intramuscular (IM) fat depots. Smith and Crouse (1984) reported that the two depots are not regulated in a coordinated manner where SC prefers acetate and IM prefers glucose, whereas Nayananjalie et al. (2015) observed no difference in substrate preference between fat depots. When fed diets based on either corn silage or ground corn, steers between treatments had no difference in marbling score, however, steers on the ground corn diets had a 52% increase in backfat thickness (Smith and Crouse 1984). Additionally, there were differences in substrate utilization by SC and IM fat depots across slaughter point in the study indicating that substrate preferences between depots may differ as total body fatness increases (Smith and Crouse (1984). In the current study steers in the 12R treatment would have been consuming a diet with 5% (DM basis) additional grass hay which replaced 5% (DM basis) dry-rolled corn; this increase in roughage in the diet would have likely resulted in an increased acetate-to-propionate ratio due to increased fermentation of the grass hay, although, no differences were observed in 12th rib fat thickness between the 12R and 7R treatment groups. In cattle, glucose availability is largely driven by metabolizable energy (ME) intake, where cattle with greater ME intake promote greater rates of gluconeogenesis (Pethick et al. 2004). Additionally, some evidence exists that supports a link between total glucose supply and IM fat deposition (Pethick et al. 1997). Gain energy density and NE_g_ intake were numerically greater in the 12R treatment, indicating that the 12R steers were consuming more total calories than the 7R treatment, which may have resulted in the increase in quality grade that was observed between the 12R and 7R treatment. Due to differences in preferential substrate utilization from SC and IM fat depots at different body fatness levels, further research is warranted to determine why differences were observed in marbling and yield grade between the 7R and 12R treatments.

Observed-to-expected NE_m_ and NE_g_ ratios for all steers in the current study were respectively 8% and 9% greater than anticipated for the study period. The steers in this study were only observed for a 28-d period prior to slaughter, thus, there are a few reasons why the observed NE values appear greater than expected. Cattle fed in current production systems are fed to higher EBF endpoints than those used to develop prediction equations (Anderson 2017). As weight increases at higher EBF percentages, it is unlikely that metabolic activity is increased by the same proportion as weight increases because adipose tissue has lower metabolic activity compared to muscle tissue (Anderson 2017). Additionally, anecdotal evidence from Anderson (2017) indicates that cattle at higher EBF percentages are more sedentary during the late finishing phase. It has been proposed that for finishing cattle at higher EBF percentage that the 0.75 exponent in the maintenance energy equation be replaced by a 0.72 exponent to adjust for the changes in cattle fatness and activity listed above (Anderson 2017). This technique was used on the steers in the current data set and the observed-to-expected NE_m_ and NE_g_ ratios were, respectively, 2% and 1% higher than expected. These results indicate that the method of adjusting the maintenance energy exponent to 0.72 improved the precision of performance prediction equations in the current study and should be further investigated by beef feedlot scientists. Increased observed-to-expected NE ratios for steers in the current study could also be explained by cyclicity of cattle growth and how the steers were gaining in the current study. Smith et al. (2022) reported that during sequential 28-35 d periods, that cattle on all historic studies evaluated had cyclic ADG with peaks and troughs compared to average of the entire finishing period. While cyclicity of growth is not fully understood, the improved growth performance during the 28-d feeding period evaluated in the current study could have been due to a peak in cyclical growth that was captured considering the small feeding window of the study.

## Conclusion

Increasing dietary roughage equivalent (or increasing NDF content from 17.5 to 20.5%) during the final 28 d prior to harvest was not an effective management strategy to reduce the severity or prevalence of liver abscesses. However, DMI was increased for the 12R steers and as a result NE_g_ intake and GED did not differ between treatments. As a result, growth performance and carcass characteristics were unaffected by treatments. This indicates that feedlot steers can maintain comparable growth on higher roughage diets for short periods. Additionally, steers on the 12R diet consumed more total forage over the feeding period, had increased rumination metrics, and improved rumen health, indicating that increasing roughage levels prior to harvest may be a strategy to improve ruminal health and reduce risk of acidosis, without compromising growth performance and carcass quality. The period of feeding of RH may be a logistically ideal time to increase roughage inclusion in diets because diets are already being changed to accommodate the feed additive. In feeding programs using the beta-ligand lubabegron, the most likely duration would be more than 42 days. Therefore, it would be useful to test how the variables evaluated in this study would behave when applying an increased NDF concentration for a longer period. Additionally, future research should investigate the effects of roughage quality, roughage NDF levels, physically effective NDF levels, and differing days of roughage inclusion prior to harvest to determine if growth and carcass performance, rumen health, and liver abscess prevalence can be improved.

## Data Availability

None of the data were deposited in an official data repository. Data can be made available upon reasonable request to the first author (FLF) or corresponding author (ZKS).

## Abbreviations

ADF: acid detergent fiber
ADG: average daily gain
BW: body weight
CON: Control
Cp: crude protein
DM: dry matter
DMI: dry matter intake
EE: ethe rextract
EG: energy for gain
EM: energy for mainteance
g: gram
G : F: gain-to-feed ratio
h: hour
kg: kilogram
L: liter
MBW: median feeding shrunk body weight
mg: milligram
mL: milliliter
NDF: neutral detergent fiber
NE: net energy
NEg: net energy for gain
NEm: net energy for maintenance
O : E: observed-to-expected
SBW: shrunk body weight
VFA: volatile fatty acid
